# Methyl 4,4′′-difluoro-5′-meth­oxy-1,1′:3′,1′′-terphenyl-4′-carboxyl­ate

**DOI:** 10.1107/S1600536811048719

**Published:** 2011-11-23

**Authors:** Hoong-Kun Fun, Tze Shyang Chia, S. Samshuddin, B. Narayana, B. K. Sarojini

**Affiliations:** a X-ray Crystallography Unit, School of Physics, Universiti Sains Malaysia, 11800 USM, Penang, Malaysia; b Department of Studies in Chemistry, Mangalore University, Mangalagangotri, Mangalore 574 199, India; c Department of Chemistry, P. A. College of Engineering, Nadupadavu, Mangalore 574 153, India

## Abstract

In the title compound, C_21_H_16_F_2_O_3_, the pendant fluoro­benzene rings form dihedral angles of 22.22 (12) and 50.74 (11)° with the central benzene ring. In the crystal, mol­ecules are linked by C—H⋯O hydrogen bonds into chains along the *a* axis. The crystal structure also features C—H⋯π inter­actions.

## Related literature

For a related structure and background to terphenyls, see: Fun *et al.* (2011 ▶). For further related structures, see: Betz *et al.* (2011*a* ▶,*b* ▶). For further synthetic details, see: Kotnis (1990 ▶). For the stability of the temperature controller used in the data collection, see: Cosier & Glazer (1986 ▶). For reference bond lengths, see: Allen *et al.* (1987 ▶).
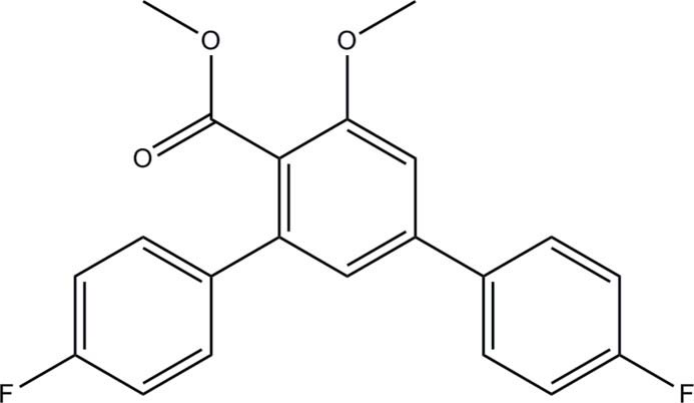


## Experimental

### 

#### Crystal data

C_21_H_16_F_2_O_3_
*M* *_r_* = 354.34
Orthorhombic, 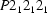

*a* = 8.1270 (1) Å
*b* = 9.4681 (1) Å
*c* = 22.3297 (3) Å
*V* = 1718.21 (4) Å^3^
*Z* = 4
Mo *K*α radiation
μ = 0.11 mm^−1^
*T* = 100 K
0.26 × 0.20 × 0.18 mm

#### Data collection

Bruker SMART APEXII CCD diffractometer
Absorption correction: multi-scan (*SADABS*; Bruker, 2009 ▶) *T*_min_ = 0.973, *T*_max_ = 0.982
11903 measured reflections
2851 independent reflections
2411 reflections with *I* > 2σ(*I*)
*R*_int_ = 0.043

#### Refinement

*R*[*F* ^2^ > 2σ(*F* ^2^)] = 0.048
*wR*(*F* ^2^) = 0.102
*S* = 1.13
2851 reflections
235 parameters
H-atom parameters constrained
Δρ_max_ = 0.51 e Å^−3^
Δρ_min_ = −0.28 e Å^−3^

### 

Data collection: *APEX2* (Bruker, 2009 ▶); cell refinement: *SAINT* (Bruker, 2009 ▶); data reduction: *SAINT*; program(s) used to solve structure: *SHELXTL* (Sheldrick, 2008 ▶); program(s) used to refine structure: *SHELXTL*; molecular graphics: *SHELXTL* software used to prepare material for publication: *SHELXTL* and *PLATON* (Spek, 2009 ▶).

## Supplementary Material

Crystal structure: contains datablock(s) global, I. DOI: 10.1107/S1600536811048719/hb6516sup1.cif

Structure factors: contains datablock(s) I. DOI: 10.1107/S1600536811048719/hb6516Isup2.hkl

Supplementary material file. DOI: 10.1107/S1600536811048719/hb6516Isup3.cml

Additional supplementary materials:  crystallographic information; 3D view; checkCIF report

## Figures and Tables

**Table 1:** Hydrogen-bond geometry (Å, °) *Cg*1 and *Cg*2 are the centroids of the C13—C18 and C7–C12 benzene rings, respectively.

*D*—H⋯*A*	*D*—H	H⋯*A*	*D*⋯*A*	*D*—H⋯*A*
C1—H1*A*⋯O2^i^	0.95	2.57	3.310 (3)	135
C1—H1*A*⋯*Cg*1^ii^	0.95	2.76	3.367 (3)	123
C19—H19*A*⋯*Cg*2^iii^	0.98	2.62	3.466 (2)	144

Symmetry codes: (i) 
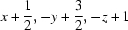
; (ii) 
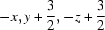
; (iii) 
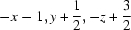
.

**Fig. 1.**
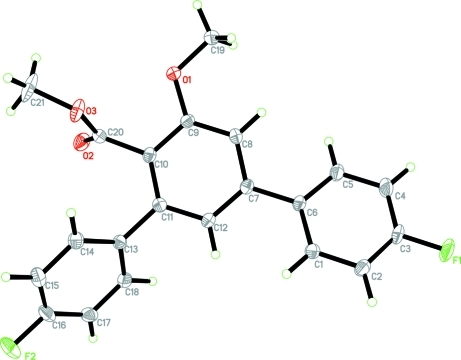
The molecular structure of the title compound with 50% probability displacement ellipsoids.

**Fig. 2.**
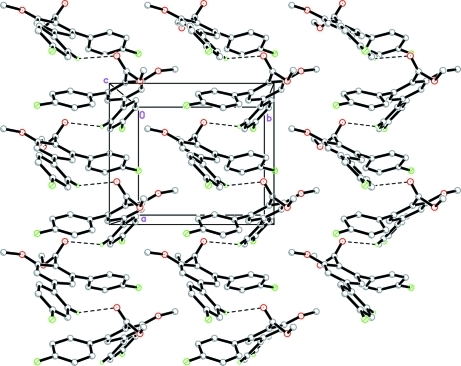
The crystal packing of the title compound. The dashed lines represent the hydrogen bonds. For clarity sake, hydrogen atoms not involved in hydrogen bonding have been omitted.

## supplementary crystallographic information

### Comment

As part of our ongonig studies of terphenyls (Fun *et al.*, 2011), the title compound was prepared and its crystal structure is reported. The precursor of the title compound was prepared from 4,4'-difluoro chalcone by several steps.

The molecular structure of the title compound is shown in Fig. 1. The least-squares planes of the two fluorophenyl rings (C1–C6 & C13–C18) make dihedral angles of 22.22 (12) and 50.74 (11)°, respectively, with the least-squares plane of the central benzene ring(C7–C12) in the terphenyl moiety. Bond lengths (Allen *et al.*, 1987) and angles are within normal ranges and are comparable to related structures (Betz *et al.*, 2011*a*,*b*).

In the crystal structure, (Fig. 2), the molecules are interconnected by C1—H1A···O2 hydrogen bonds (Table 1) into infinite chains along *a* axis. The crystal structure is further stabilized by C—H···π interactions, involving the centroids of C7–C12 and C13—C18 benzene rings.

### Experimental

The title compound was prepared by the aromatization of a cyclohexenone derivative, methyl 4,6-bis(4-fluorophenyl)-2-oxocyclohex-3-ene-1-carboxylate, using iodine and methanol at reflux condition (Kotnis, 1990). Colourless blocks of (I) were grown from methanol by slow evaporation method (*m.p.*: 401 K).

### Refinement

All H atoms were positioned geometrically [C—H = 0.95 or 0.98 Å] and refined using a riding model with *U*_iso_(H) = 1.2 or 1.5*U*_eq_(C).

### Crystal data

**Table:**

C_21_H_16_F_2_O_3_	*F*(000) = 736
*M**_r_* = 354.34	*D*_x_ = 1.370 Mg m^−^^3^
Orthorhombic, *P*2_1_2_1_2_1_	Mo *K*α radiation, λ = 0.71073 Å
Hall symbol: P 2ac 2ab	Cell parameters from 3629 reflections
*a* = 8.1270 (1) Å	θ = 2.8–30.6°
*b* = 9.4681 (1) Å	µ = 0.11 mm^−^^1^
*c* = 22.3297 (3) Å	*T* = 100 K
*V* = 1718.21 (4) Å^3^	Block, colourless
*Z* = 4	0.26 × 0.20 × 0.18 mm

### Data collection

**Table:**

Bruker SMART APEXII CCD diffractometer	2851 independent reflections
Radiation source: fine-focus sealed tube	2411 reflections with *I* > 2σ(*I*)
graphite	*R*_int_ = 0.043
φ and ω scans	θ_max_ = 30.0°, θ_min_ = 2.3°
Absorption correction: multi-scan (*SADABS*; Bruker, 2009)	*h* = −11→9
*T*_min_ = 0.973, *T*_max_ = 0.982	*k* = −13→10
11903 measured reflections	*l* = −31→30

### Refinement

**Table:**

Refinement on *F*^2^	Primary atom site location: structure-invariant direct methods
Least-squares matrix: full	Secondary atom site location: difference Fourier map
*R*[*F*^2^ > 2σ(*F*^2^)] = 0.048	Hydrogen site location: inferred from neighbouring sites
*wR*(*F*^2^) = 0.102	H-atom parameters constrained
*S* = 1.13	*w* = 1/[σ^2^(*F*_o_^2^) + (0.0345*P*)^2^ + 0.548*P*] where *P* = (*F*_o_^2^ + 2*F*_c_^2^)/3
2851 reflections	(Δ/σ)_max_ < 0.001
235 parameters	Δρ_max_ = 0.51 e Å^−^^3^
0 restraints	Δρ_min_ = −0.28 e Å^−^^3^

### Special details

**Table:**

Experimental. The crystal was placed in the cold stream of an Oxford Cryosystems Cobra open-flow nitrogen cryostat (Cosier & Glazer, 1986) operating at 100.0 (1) K.
Geometry. All e.s.d.'s (except the e.s.d. in the dihedral angle between two l.s. planes) are estimated using the full covariance matrix. The cell e.s.d.'s are taken into account individually in the estimation of e.s.d.'s in distances, angles and torsion angles; correlations between e.s.d.'s in cell parameters are only used when they are defined by crystal symmetry. An approximate (isotropic) treatment of cell e.s.d.'s is used for estimating e.s.d.'s involving l.s. planes.
Refinement. Refinement of *F*^2^ against ALL reflections. The weighted *R*-factor *wR* and goodness of fit *S* are based on *F*^2^, conventional *R*-factors *R* are based on *F*, with *F* set to zero for negative *F*^2^. The threshold expression of *F*^2^ > σ(*F*^2^) is used only for calculating *R*-factors(gt) *etc*. and is not relevant to the choice of reflections for refinement. *R*-factors based on *F*^2^ are statistically about twice as large as those based on *F*, and *R*- factors based on ALL data will be even larger.

### Fractional atomic coordinates and isotropic or equivalent isotropic displacement parameters (Å^2^)

**Table:**

	*x*	*y*	*z*	*U*_iso_*/*U*_eq_	
F1	0.7209 (2)	0.59514 (17)	0.82984 (6)	0.0346 (4)	
F2	0.5985 (2)	1.05766 (16)	0.32763 (7)	0.0340 (4)	
O1	0.2900 (2)	0.27202 (16)	0.49542 (7)	0.0214 (4)	
O2	0.1948 (2)	0.54018 (18)	0.39484 (7)	0.0252 (4)	
O3	0.3970 (2)	0.38062 (19)	0.38201 (7)	0.0264 (4)	
C1	0.6798 (3)	0.6457 (2)	0.66999 (10)	0.0182 (5)	
H1A	0.7260	0.7022	0.6391	0.022*	
C2	0.7354 (3)	0.6627 (3)	0.72845 (10)	0.0218 (5)	
H2A	0.8180	0.7301	0.7379	0.026*	
C3	0.6670 (3)	0.5787 (3)	0.77222 (10)	0.0223 (5)	
C4	0.5468 (4)	0.4812 (3)	0.76107 (10)	0.0260 (6)	
H4A	0.5018	0.4255	0.7924	0.031*	
C5	0.4921 (3)	0.4661 (3)	0.70215 (10)	0.0234 (5)	
H5A	0.4087	0.3990	0.6934	0.028*	
C6	0.5580 (3)	0.5481 (2)	0.65562 (9)	0.0163 (4)	
C7	0.4997 (3)	0.5310 (2)	0.59270 (9)	0.0155 (4)	
C8	0.4251 (3)	0.4044 (2)	0.57459 (9)	0.0173 (5)	
H8A	0.4136	0.3287	0.6022	0.021*	
C9	0.3682 (3)	0.3896 (2)	0.51633 (10)	0.0160 (4)	
C10	0.3870 (3)	0.4985 (2)	0.47469 (9)	0.0160 (4)	
C11	0.4653 (3)	0.6234 (2)	0.49160 (9)	0.0153 (4)	
C12	0.5185 (3)	0.6392 (2)	0.55078 (10)	0.0162 (4)	
H12A	0.5686	0.7254	0.5627	0.019*	
C13	0.4995 (3)	0.7377 (2)	0.44754 (10)	0.0162 (4)	
C14	0.5776 (3)	0.7085 (2)	0.39312 (10)	0.0202 (5)	
H14A	0.6068	0.6140	0.3836	0.024*	
C15	0.6131 (3)	0.8163 (3)	0.35290 (11)	0.0236 (5)	
H15A	0.6665	0.7966	0.3160	0.028*	
C16	0.5690 (3)	0.9521 (3)	0.36781 (11)	0.0234 (5)	
C17	0.4933 (3)	0.9866 (2)	0.42104 (11)	0.0223 (5)	
H17A	0.4643	1.0815	0.4300	0.027*	
C18	0.4605 (3)	0.8777 (2)	0.46129 (10)	0.0202 (5)	
H18A	0.4109	0.8990	0.4987	0.024*	
C19	0.2657 (3)	0.1589 (2)	0.53712 (10)	0.0207 (5)	
H19A	0.2153	0.0784	0.5165	0.031*	
H19B	0.1932	0.1906	0.5695	0.031*	
H19C	0.3721	0.1303	0.5539	0.031*	
C20	0.3146 (3)	0.4776 (2)	0.41326 (10)	0.0181 (5)	
C21	0.3284 (4)	0.3466 (4)	0.32363 (12)	0.0410 (8)	
H21A	0.2347	0.2825	0.3286	0.062*	
H21B	0.4127	0.3009	0.2990	0.062*	
H21C	0.2915	0.4336	0.3040	0.062*	

### Atomic displacement parameters (Å^2^)

**Table:**

	*U*^11^	*U*^22^	*U*^33^	*U*^12^	*U*^13^	*U*^23^
F1	0.0421 (10)	0.0490 (10)	0.0127 (7)	−0.0089 (8)	−0.0074 (7)	−0.0038 (7)
F2	0.0373 (10)	0.0306 (8)	0.0341 (8)	−0.0019 (8)	0.0038 (8)	0.0178 (7)
O1	0.0310 (10)	0.0176 (7)	0.0157 (7)	−0.0079 (7)	−0.0028 (7)	0.0001 (6)
O2	0.0299 (10)	0.0233 (8)	0.0224 (8)	0.0058 (8)	−0.0088 (8)	−0.0020 (7)
O3	0.0301 (10)	0.0354 (10)	0.0137 (8)	0.0092 (8)	−0.0040 (7)	−0.0068 (7)
C1	0.0211 (12)	0.0182 (10)	0.0152 (10)	−0.0020 (10)	0.0016 (9)	−0.0008 (8)
C2	0.0232 (13)	0.0239 (11)	0.0184 (11)	−0.0035 (10)	−0.0031 (10)	−0.0035 (9)
C3	0.0268 (13)	0.0292 (12)	0.0109 (10)	0.0017 (11)	−0.0042 (10)	−0.0036 (9)
C4	0.0308 (15)	0.0330 (13)	0.0141 (10)	−0.0048 (12)	0.0007 (10)	0.0033 (10)
C5	0.0255 (13)	0.0292 (12)	0.0154 (10)	−0.0076 (11)	−0.0023 (10)	0.0014 (10)
C6	0.0182 (11)	0.0182 (10)	0.0125 (9)	0.0006 (9)	0.0000 (9)	−0.0017 (8)
C7	0.0140 (10)	0.0183 (10)	0.0141 (10)	0.0009 (9)	−0.0003 (9)	−0.0016 (8)
C8	0.0198 (12)	0.0184 (10)	0.0136 (10)	−0.0010 (9)	0.0007 (9)	0.0021 (8)
C9	0.0162 (11)	0.0156 (9)	0.0161 (10)	−0.0006 (8)	−0.0016 (9)	−0.0012 (8)
C10	0.0167 (11)	0.0176 (10)	0.0136 (9)	0.0021 (8)	0.0004 (9)	−0.0013 (8)
C11	0.0158 (11)	0.0147 (9)	0.0154 (10)	0.0022 (8)	0.0012 (9)	0.0000 (8)
C12	0.0172 (11)	0.0168 (10)	0.0146 (10)	−0.0001 (9)	−0.0005 (9)	−0.0017 (8)
C13	0.0158 (11)	0.0176 (10)	0.0151 (10)	−0.0007 (9)	−0.0025 (9)	0.0013 (8)
C14	0.0203 (12)	0.0210 (11)	0.0192 (10)	0.0012 (10)	−0.0026 (10)	0.0018 (9)
C15	0.0223 (13)	0.0302 (13)	0.0183 (11)	−0.0001 (10)	0.0016 (10)	0.0048 (10)
C16	0.0230 (13)	0.0229 (11)	0.0244 (12)	−0.0037 (10)	−0.0019 (10)	0.0106 (10)
C17	0.0253 (13)	0.0161 (10)	0.0255 (12)	−0.0011 (10)	−0.0048 (11)	0.0027 (9)
C18	0.0252 (13)	0.0176 (10)	0.0179 (11)	−0.0003 (9)	−0.0027 (10)	0.0001 (9)
C19	0.0237 (13)	0.0189 (10)	0.0197 (11)	−0.0042 (10)	−0.0022 (10)	0.0009 (9)
C20	0.0222 (12)	0.0158 (10)	0.0163 (10)	−0.0024 (9)	−0.0002 (9)	0.0014 (8)
C21	0.052 (2)	0.0546 (18)	0.0166 (12)	0.0205 (17)	−0.0117 (13)	−0.0164 (13)

### Geometric parameters (Å, °)

**Table:**

F1—C3	1.368 (3)	C9—C10	1.397 (3)
F2—C16	1.364 (3)	C10—C11	1.395 (3)
O1—C9	1.364 (3)	C10—C20	1.506 (3)
O1—C19	1.433 (3)	C11—C12	1.398 (3)
O2—C20	1.211 (3)	C11—C13	1.489 (3)
O3—C20	1.334 (3)	C12—H12A	0.9500
O3—C21	1.454 (3)	C13—C18	1.397 (3)
C1—C2	1.391 (3)	C13—C14	1.399 (3)
C1—C6	1.392 (3)	C14—C15	1.390 (3)
C1—H1A	0.9500	C14—H14A	0.9500
C2—C3	1.377 (3)	C15—C16	1.376 (3)
C2—H2A	0.9500	C15—H15A	0.9500
C3—C4	1.367 (4)	C16—C17	1.378 (3)
C4—C5	1.396 (3)	C17—C18	1.393 (3)
C4—H4A	0.9500	C17—H17A	0.9500
C5—C6	1.403 (3)	C18—H18A	0.9500
C5—H5A	0.9500	C19—H19A	0.9800
C6—C7	1.491 (3)	C19—H19B	0.9800
C7—C12	1.396 (3)	C19—H19C	0.9800
C7—C8	1.403 (3)	C21—H21A	0.9800
C8—C9	1.388 (3)	C21—H21B	0.9800
C8—H8A	0.9500	C21—H21C	0.9800
			
C9—O1—C19	116.85 (17)	C7—C12—H12A	119.3
C20—O3—C21	115.4 (2)	C11—C12—H12A	119.3
C2—C1—C6	121.6 (2)	C18—C13—C14	118.8 (2)
C2—C1—H1A	119.2	C18—C13—C11	120.1 (2)
C6—C1—H1A	119.2	C14—C13—C11	121.00 (19)
C3—C2—C1	117.9 (2)	C15—C14—C13	120.7 (2)
C3—C2—H2A	121.0	C15—C14—H14A	119.7
C1—C2—H2A	121.0	C13—C14—H14A	119.7
C4—C3—F1	118.5 (2)	C16—C15—C14	118.4 (2)
C4—C3—C2	123.3 (2)	C16—C15—H15A	120.8
F1—C3—C2	118.2 (2)	C14—C15—H15A	120.8
C3—C4—C5	117.9 (2)	F2—C16—C15	118.7 (2)
C3—C4—H4A	121.0	F2—C16—C17	118.2 (2)
C5—C4—H4A	121.0	C15—C16—C17	123.1 (2)
C4—C5—C6	121.3 (2)	C16—C17—C18	117.8 (2)
C4—C5—H5A	119.3	C16—C17—H17A	121.1
C6—C5—H5A	119.3	C18—C17—H17A	121.1
C1—C6—C5	117.9 (2)	C17—C18—C13	121.1 (2)
C1—C6—C7	121.0 (2)	C17—C18—H18A	119.4
C5—C6—C7	121.1 (2)	C13—C18—H18A	119.4
C12—C7—C8	118.75 (19)	O1—C19—H19A	109.5
C12—C7—C6	121.2 (2)	O1—C19—H19B	109.5
C8—C7—C6	120.1 (2)	H19A—C19—H19B	109.5
C9—C8—C7	120.0 (2)	O1—C19—H19C	109.5
C9—C8—H8A	120.0	H19A—C19—H19C	109.5
C7—C8—H8A	120.0	H19B—C19—H19C	109.5
O1—C9—C8	124.0 (2)	O2—C20—O3	124.2 (2)
O1—C9—C10	115.18 (18)	O2—C20—C10	124.0 (2)
C8—C9—C10	120.9 (2)	O3—C20—C10	111.8 (2)
C9—C10—C11	119.69 (19)	O3—C21—H21A	109.5
C9—C10—C20	117.84 (19)	O3—C21—H21B	109.5
C11—C10—C20	122.42 (19)	H21A—C21—H21B	109.5
C10—C11—C12	119.20 (19)	O3—C21—H21C	109.5
C10—C11—C13	121.49 (19)	H21A—C21—H21C	109.5
C12—C11—C13	119.26 (19)	H21B—C21—H21C	109.5
C7—C12—C11	121.4 (2)		
			
C6—C1—C2—C3	0.5 (4)	C9—C10—C11—C13	175.2 (2)
C1—C2—C3—C4	−0.7 (4)	C20—C10—C11—C13	−7.3 (3)
C1—C2—C3—F1	−179.9 (2)	C8—C7—C12—C11	−0.2 (4)
F1—C3—C4—C5	179.6 (2)	C6—C7—C12—C11	179.5 (2)
C2—C3—C4—C5	0.4 (4)	C10—C11—C12—C7	2.1 (3)
C3—C4—C5—C6	0.0 (4)	C13—C11—C12—C7	−175.4 (2)
C2—C1—C6—C5	−0.1 (4)	C10—C11—C13—C18	132.5 (2)
C2—C1—C6—C7	−179.9 (2)	C12—C11—C13—C18	−50.0 (3)
C4—C5—C6—C1	−0.2 (4)	C10—C11—C13—C14	−50.5 (3)
C4—C5—C6—C7	179.7 (2)	C12—C11—C13—C14	127.0 (2)
C1—C6—C7—C12	−22.6 (3)	C18—C13—C14—C15	−1.3 (4)
C5—C6—C7—C12	157.5 (2)	C11—C13—C14—C15	−178.3 (2)
C1—C6—C7—C8	157.1 (2)	C13—C14—C15—C16	−0.2 (4)
C5—C6—C7—C8	−22.8 (3)	C14—C15—C16—F2	−177.8 (2)
C12—C7—C8—C9	−1.5 (3)	C14—C15—C16—C17	0.8 (4)
C6—C7—C8—C9	178.8 (2)	F2—C16—C17—C18	178.7 (2)
C19—O1—C9—C8	0.4 (3)	C15—C16—C17—C18	0.0 (4)
C19—O1—C9—C10	−178.9 (2)	C16—C17—C18—C13	−1.6 (4)
C7—C8—C9—O1	−178.0 (2)	C14—C13—C18—C17	2.2 (4)
C7—C8—C9—C10	1.3 (4)	C11—C13—C18—C17	179.2 (2)
O1—C9—C10—C11	179.9 (2)	C21—O3—C20—O2	−3.3 (3)
C8—C9—C10—C11	0.6 (3)	C21—O3—C20—C10	175.9 (2)
O1—C9—C10—C20	2.4 (3)	C9—C10—C20—O2	108.9 (3)
C8—C9—C10—C20	−177.0 (2)	C11—C10—C20—O2	−68.6 (3)
C9—C10—C11—C12	−2.3 (3)	C9—C10—C20—O3	−70.2 (3)
C20—C10—C11—C12	175.2 (2)	C11—C10—C20—O3	112.3 (2)

### Hydrogen-bond geometry (Å, °)

**Table:**

Cg1 and Cg2 are the centroids of the C13—C18 and C7–C12 benzene rings, respectively.

**Table:**

*D*—H···*A*	*D*—H	H···*A*	*D*···*A*	*D*—H···*A*
C1—H1A···O2^i^	0.95	2.57	3.310 (3)	135
C1—H1A···Cg1^ii^	0.95	2.76	3.367 (3)	123
C19—H19A···Cg2^iii^	0.98	2.62	3.466 (2)	144

Symmetry codes: (i) *x*+1/2, −*y*+3/2, −*z*+1; (ii) −*x*, *y*+3/2, −*z*+3/2; (iii) −*x*−1, *y*+1/2, −*z*+3/2.
